# Noradrenergic innervation across brain regions is altered by aging and by disease progression in a mouse model of Alzheimer’s disease neuropathology

**DOI:** 10.1371/journal.pone.0340611

**Published:** 2026-02-18

**Authors:** Nicole M. Hernandez, Manuel Silva-Pérez, Jeannie Chin

**Affiliations:** 1 Department of Neuroscience, Baylor College of Medicine, Houston, Texas, United States of America; 2 Center for Comparative Medicine, Baylor College of Medicine, Houston, Texas, United States of America; Museo Storico della Fisica e Centro Studi e Ricerche Enrico Fermi, ITALY

## Abstract

Norepinephrine plays critical roles in modulating arousal and attention, is highly dynamic in awake, behaving individuals, and has anti-inflammatory and neuroprotective actions. Notably, the locus coeruleus (LC), the primary source of norepinephrine in the central nervous system, is among the first brain regions to show pathological alterations in early stages of Alzheimer’s disease (AD). LC neuronal loss and associated reductions in norepinephrine in the brain have therefore been postulated to play a key role in AD pathophysiology. LC neurons and their axons have been studied in several mouse models of AD-related neuropathology to investigate their contribution to brain dysfunction in AD. However, the time course and spatial distribution of alterations in noradrenergic (norepinephrine-containing) LC projections are not fully understood. We therefore evaluated the density of noradrenergic axonal projections in the cortex and across subregions of the hippocampus in transgenic mice expressing mutant human amyloid precursor protein (APP) and in nontransgenic wild-type littermate controls at 2, 6, 12 and 20 months of age. In comparison to age-matched controls, APP mice displayed region-specific alterations in hippocampal noradrenergic fiber density that followed distinct age-related trajectories, along with subtle decreases in cortical noradrenergic fiber density. The alterations in noradrenergic innervation in APP mice were not associated with the extent of amyloid-β (Aβ) plaque load in the hippocampus or cortex and occurred in the absence of neuronal loss or Aβ plaques in the LC. In wild-type mice, there were subtle but robust alterations in noradrenergic fiber density across the brain between 2–20 months of age. These results reveal the presence of spatiotemporally complex alterations in noradrenergic innervation in the brain across both normal aging and disease progression.

## Introduction

The locus coeruleus (LC) is the main source of noradrenergic projections carrying norepinephrine in the brain. The LC-norepinephrine system plays critical roles in attention [1], memory [1], brain connectivity [2], and critical homeostatic processes such as modulating inflammation [3–5], controlling sleep [6], and optimizing the active regulation and redistribution of blood flow to dynamically meet oxygen demands in active brain areas [7]. Notably, the LC is among the first brain structures to undergo pathological changes in Alzheimer’s disease (AD) [8] and is subject to neurodegeneration throughout the disease course [9–14].

Many pieces of evidence highlight the relevance of noradrenergic neurotransmission in age- and disease-related brain function and dysfunction. Higher integrity of the LC, visualized by magnetic resonance imaging, was positively associated with cortical thickness [15] and memory [16] in older adults, and higher brain norepinephrine concentration corresponded with better Mini-Mental State Examination scores in individuals with AD [17]. Conversely, low neuronal density in the LC is associated with accelerated cognitive decline and memory deficits [18,19], and with the manifestation of neuropsychiatric symptoms in AD patients, such as hallucinations, agitation, depression, apathy, and motor disturbances [20]. Thus, noradrenergic dysfunction may contribute to AD pathophysiology [21].

LC cell loss is often already present in individuals with mild cognitive impairment (MCI) and becomes more pronounced in individuals with AD [22], in whom the magnitude of cell loss corresponds with the duration of disease [12,13] and with Braak stage [23]. Cell loss is typically accompanied by alterations in the density and morphology of noradrenergic axons in the hippocampus [24,25] and by reductions in norepinephrine content in several brain areas including the frontal and temporal lobes, prefrontal cortex, cingulate gyrus, hippocampus, caudate nucleus, amygdala, thalamus, and hypothalamus [10,26–31].

Data from animal models have also highlighted the importance of the LC-norepinephrine system in AD. Similar to humans with AD, several amyloid precursor protein (APP) mouse models of AD neuropathology exhibit a loss of LC neurons with age [32–36]. Decreases in noradrenergic axonal density in the hippocampus and cortex in APP mice have also been described [34,37]. Experimental reduction of norepinephrine levels in APP mice has been shown to exacerbate inflammation and accumulation of amyloid-β (Aβ) plaques [38,39], further highlighting the link between the LC-norepinephrine system and disease progression. Noradrenergic depletion increased cognitive deficits in APP/PS1 mice [40], whereas chronic enhancement of norepinephrine levels improved learning in 5xFAD mice [41]. The deleterious effects of noradrenergic depletion have also been demonstrated in mouse models of tau pathology [42]. Reductions in noradrenergic axon density and norepinephrine content have also been reported in APP rats and were accompanied by learning deficits that were rescued by chemogenetic stimulation of the LC [43]. The LC-norepinephrine system is a growing area of AD research, and many studies have demonstrated the importance of the LC-norepinephrine system in AD.

Previous reports have yielded interesting results regarding norepinephrine innervation within particular neuroanatomical regions at specific timepoints. However, the precise temporal and spatial dynamics of anatomical alterations of the LC-norepinephrine system are not well understood. We addressed this gap in knowledge by characterizing regional changes in noradrenergic axon density in subregions of the hippocampus as well as in the cortex throughout multiple age- and disease-progression states. To do this, we utilized an APP mouse model of AD-related neuropathology (line J20). In this model, robust subclinical epileptiform activity and alterations in sleep stability and architecture are present by 2 months of age [44,45], and cognitive deficits are present by 3–4 months of age [46]. These alterations precede Aβ plaque deposition, which begins around 6 months of age [47]. We therefore studied APP mice and nontransgenic (NTG) littermate controls at 2, 6, 12, and 20 months of age to span the course of aging and disease progression. We found consistent alterations in noradrenergic innervation that were highly region- and age-specific and were not accompanied by cell loss in the LC. Aβ plaque load in the hippocampus and cortex did not predict the magnitude of noradrenergic axon density changes, and we found no Aβ plaques in the LC or neighboring brainstem regions. This study provides a valuable resource of AD-related alterations in the LC-norepinephrine system that occur in an age- and disease-specific manner.

## Materials and methods

### Mice

This study was carried out using heterozygous transgenic mice expressing human amyloid precursor protein (APP) carrying the Swedish (K670N, M671L) and Indiana (V717F) familial AD mutations (Line J20; MMRRC_034836-JAX; hAPP770 transgene) [47]. This line was backcrossed onto a C57BL/6J background (The Jackson Laboratory) for over ten generations, and heterozygosity was maintained by breeding APP mice with wild-type C57BL/6J mice from the Jackson Laboratory. Control groups consisted of age- and sex-matched nontransgenic (NTG) wild-type mice from the same line. Mice were group-housed in cages with pelleted cellulose bedding, EnviroPak nestlets, and *ad libitum* access to feed (5V5R; LabDiet) and water. Temperature was kept at 22 ± 1°C and a 12:12 light-dark cycle was maintained. Prior to tissue harvest, mice were individually housed and moved to a quiet environment for two days.

Brains from APP and NTG mice were harvested at different timepoints between 2 and 20 months of age. For tissue collection, euthanasia solution (EUTHASOL, Virbac) was administered intraperitoneally, and transcardial perfusion with ice-cold normal saline solution was performed. Brains were extracted and hemisected. Hemibrains were post-fixed in 4% phosphate-buffered paraformaldehyde at 4°C for two days for immunohistochemistry.

This study was performed in accordance with recommendations in the Guide for the Care and Use of Laboratory Animals of the National Institutes of Health, under protocol AN-6943 as approved by the Baylor College of Medicine Institutional Animal Care and Use Committee.

### Immunohistochemical experiments

Tissue preparation and immunohistochemistry were performed as previously described [44,45,48–52]. Paraformaldehyde-fixed hemibrains used for immunohistochemistry were cryoprotected by incubation in a 30% sucrose solution in PBS at 4°C for two days and then sectioned coronally at a thickness of 30 μm using a freezing, sliding microtome (HM 450, Microm). The sections were distributed into ten subseries, each containing every tenth section throughout the rostral-caudal extent of the brain, and sections were stored at −20°C in cryoprotectant solution (30% glycerol, 30% ethylene glycol, 40% PBS) until use. Each immunostain was carried out using one subseries of free-floating sections, except for locus coeruleus (LC) cell count stains, for which two equidistant subseries were combined to achieve a resolution of one section every 150 µm instead of every 300 µm.

For immunohistochemical staining using 3,3-diaminobenzidine (DAB) as the chromogen, antibodies included: mouse anti-norepinephrine transporter primary (1:250; monoclonal; catalog # MA5–24647, Invitrogen), mouse anti-tyrosine hydroxylase primary (1:5000; monoclonal; catalog # 22941, ImmunoStar), rabbit anti-amyloid-β primary (1:1000; polyclonal; catalog # 71–5800, Invitrogen), donkey anti-mouse secondary (1:500; polyclonal; catalog # 715-065-150, Jackson ImmunoResearch), and goat anti-rabbit secondary (1:200; clonal; catalog # 31820, Vector Laboratories), with enhanced signal amplification using Avidin-Biotin Complex (Vector Laboratories). For immunofluorescence, primary antibodies included: mouse anti-norepinephrine transporter (1:300; catalog # MA5–24647, Invitrogen), mouse anti-tyrosine hydroxylase (1:2000; catalog # 22941, ImmunoStar), rabbit anti-GAP-43 (1:1000; catalog # NB300–143, Novus Biologicals), and rabbit anti-amyloid-β (1:300; catalog # 71–5800, Invitrogen). Secondary antibodies included: goat anti-mouse AlexaFluor 488 secondary (1:500; catalog # A-11029, Invitrogen) and goat anti-rabbit AlexaFluor 594 secondary (1:500; catalog # A-11037, Invitrogen). Prior to immunostaining against Aβ, brain sections were subjected to antigen retrieval by incubation in a 90% formic acid solution at room temperature for 1 minute.

Unless otherwise stated, immunohistochemistry experiments were performed on tissue from mice at four different ages: 2 months (11 NTG: 6 females, 5 males; 12 APP: 6 females, 6 males), 6 months (11 NTG: 6 females, 5 males; 12 APP: 7 females, 5 males), 12 months (11 NTG: 6 females, 5 males; 11 APP: 5 females, 6 males), and 20 months (11 NTG: 6 females, 5 males; 12 APP: 6 females, 6 males). For all methods of data analysis described below, the investigator performing the analyses was blinded with respect to the genotype and sex of the mice being analyzed.

## Data analysis

### Quantification of noradrenergic axon density

The density of norepinephrine transporter (NET)-positive noradrenergic axons was measured using the Trainable Weka Segmentation [53] plugin included in Fiji (ImageJ) [54,55]. Briefly, a machine learning classifier was trained on images of mouse brain cortex and hippocampus immunostained for NET to automatically detect noradrenergic axons. An investigator manually delimitated multiple instances of NET-positive axons and axon-free “empty” space, using these selections to train the classifier. The classifier was further refined with additional selections until all axons were precisely traced, and all empty space and high background areas were ignored. The classifier was then saved and applied to all samples without further modification. The resulting “segmentation maps” were evaluated and analyzed.

Noradrenergic axon density was quantified for the cortex and the following hippocampal regions, at approximately 2.06 mm posterior to Bregma: hilus, granule cell layer (GCL), molecular layer (ML), stratum lacunosum-moleculare (SLM), CA1, and CA3. Density was calculated as percent area covered by NET-positive projections. In all cases, analysis was carried out on two brain sections per mouse and averaged.

### Skeleton analysis of noradrenergic axons

Fiji’s Skeletonize (2D/3D) plugin was used to quantify total axon length. The previously described segmentation maps were used as the input. The Skeletonize (2D/3D) plugin selected the area classified as covered by noradrenergic axons and generated a skeleton of uniform thickness that traced the whole length of the axons, irrespective of axon thickness. We calculated the percent area covered by the skeleton. Analysis was performed using two brain sections per mouse (approximately 2.06 mm posterior to Bregma) and the results were averaged.

### LC cell counts

Two brain sections immunostained against tyrosine hydroxylase (TH) were quantified per mouse, and the total number of cells counted in both sections was calculated. Mice whose LC sections did not match the general anterior-posterior location of the other individuals (approximately 5.40 mm and 5.68 mm posterior to Bregma), or that only showed one matching brain section, were excluded from the study.

### Assessment of area covered by amyloid-β plaques

The percent area covered by plaques was assessed in the cortex and hippocampus of 12- and 20-month-old APP mice through particle analysis in Fiji. A threshold was established that highlighted the extent of Aβ plaques, leaving out intracellular Aβ and other unrelated structures. The threshold was then kept constant throughout all samples and the area fraction occupied by staining above threshold was measured. Analysis was performed using two brain sections per mouse (at approximately 2.06 mm posterior to Bregma) and the result was averaged.

### Statistics

Data from male and female mice were analyzed separately and in combination to determine whether there were any sex-specific effects. Because both sexes exhibited similar genotype-dependent alterations and no overt sex-specific patterns were found, the data from combined sexes is presented in the figures. Differently colored dots on graphs indicate data points from male mice (black dots) or female mice (red dots). The results of all statistical analyses, including data from individual and combined sexes, can be found in S1 Datasheet. Statistical analyses were performed using Prism 10.3 (GraphPad). Unless otherwise indicated, results are reported as sample mean ± standard error of the mean. For comparison of group means, sample normality was verified using the Shapiro-Wilk test. The means of two groups were compared with unpaired two-tailed t-tests or Mann-Whitney tests. The means of multiple groups were compared using ordinary one-way ANOVA with Holm-Šídák correction for multiple comparisons. Simple linear regression analyses were carried out to evaluate the relationship between area covered by Aβ plaques and NET-positive axon density in the hippocampus and cortex. An alpha level of p < 0.05 was used as the threshold for significance.

## Results

### Noradrenergic axon density is altered in an age- and region-specific manner in APP mice

The LC is among the first brain structures to show pathological changes in MCI and AD [8,22], and LC-derived noradrenergic axons exhibit decreased density and altered morphology in the hippocampus of patients with AD [24,25]. Several mouse models of AD-related neuropathology also have documented reductions in noradrenergic fiber density in various regions of the hippocampus and cortex at specific ages [34,37]. Given the complexity of AD pathogenesis and the need for characterization of noradrenergic dysfunction throughout the disease process, we performed a detailed analysis of spatial and age-related changes in the density of hippocampal and cortical noradrenergic axons in APP mice.

**Fig 1:**
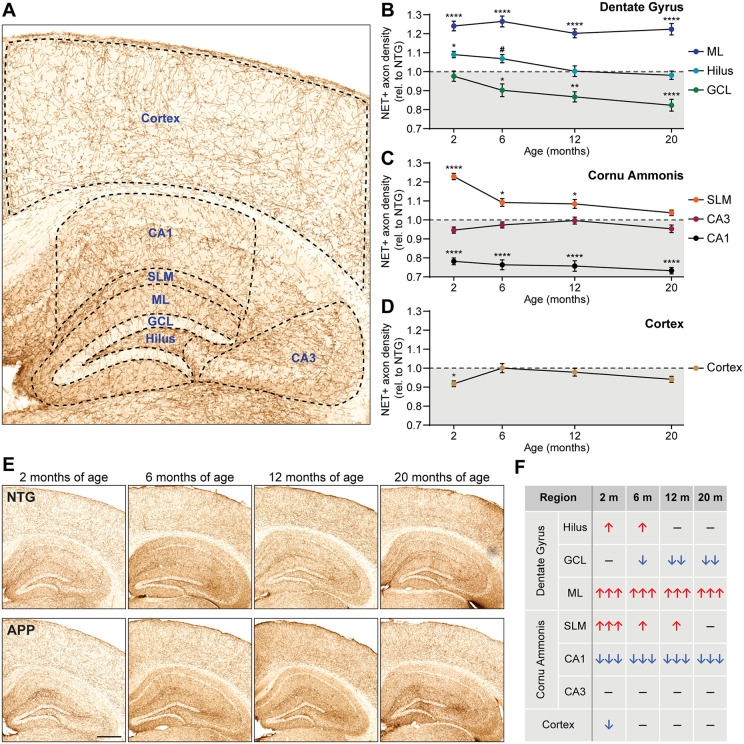
Noradrenergic axon density is altered in a region- and age-specific manner in APP mice. (A) Norepinephrine transporter (NET) immunostain of mouse cortex and hippocampus, depicting the regions that were selected for quantification of the area covered by noradrenergic axons. (B-D) Fold change of noradrenergic axon density in APP mice relative to NTG levels in the dentate gyrus (B) and Cornu Ammonis (C) of the hippocampus, and in the cortex (D), at 2, 6, 12, and 20 months of age (n = 10–12 mice per genotype and age). Values below 1 indicate reduced area covered by noradrenergic axons in APP mice with respect to the age-matched NTG groups, and values above 1 indicate increased area covered in APP with respect to NTG levels. (E) Representative examples illustrating density of NET-positive noradrenergic axons in the hippocampus and cortex of NTG and APP mice at 2, 6, 12, and 20 months of age. Scale bar, 500 µm. (F) Table summarizing direction and magnitude of changes in noradrenergic axon density in APP mice with respect to NTG mice across the different ages. ↑ , 5–12% increase; ↑↑, 12–19% increase; ↑↑↑, 19–27% increase; ↓ , 5–12% decrease; ↓↓, 12–19% decrease; ↓↓↓, 19–27% decrease; –, no significant differences. GCL, granule cell layer; ML, molecular layer; SLM, stratum lacunosum-moleculare. *, p < 0.05; **, p < 0.01; ***, p < 0.001; ****, p < 0.0001; #, p = 0.0630; one-way ANOVA with Holm-Šídák correction for multiple comparisons. Error bars indicate mean ± SEM. ANOVA results are the following: ML, [F = 28.23, p < 0.0001]; hilus, [F = 3.65, p = 0.0018]; GCL, [F = 7.05, p < 0.0001]; SLM, [F = 13.71, p < 0.0001]; CA3, [F = 1.56, p = 0.1601]; CA1, [F = 33.61, p < 0.0001]; cortex, [F = 2.28, p = 0.0360].

We assessed noradrenergic axon density in the hippocampus and cortex of APP mice and age-matched NTG mice at 2, 6, 12 and 20 months of age. Because the hippocampus has a high degree of spatial heterogeneity, we examined axon density in the hilus, granule cell layer (GCL), molecular layer (ML), stratum lacunosum-moleculare (SLM), CA1, and CA3, as shown in Fig 1A. Noradrenergic axon density was markedly increased in the ML of APP mice across all age groups whereas the hilus showed a subtle increase at 2 and 6 months of age (Fig 1B, E). The GCL of APP mice showed a decrease in noradrenergic axon density that was apparent at 6 months of age and further progressed at 12 and 20 months of age (Fig 1B, E). The SLM showed a marked increase in axon density in 2-month-old APP mice that remained elevated at 6 and 12 months of age (Fig 1C, E). Noradrenergic axon density in CA1 was robustly and consistently decreased in APP mice with respect to NTG mice at all timepoints, whereas CA3 axon density did not change significantly (Fig 1C, E).

We also analyzed noradrenergic axon density in cortical areas. We found an initial decrease in APP mice at 2 months of age that was no longer present by 6 months of age, and a trend towards reduced density at 20 months of age (Fig 1D, E). The direction and magnitude of changes in noradrenergic axon density in each brain area are summarized in Fig 1F.

**Fig 2:**
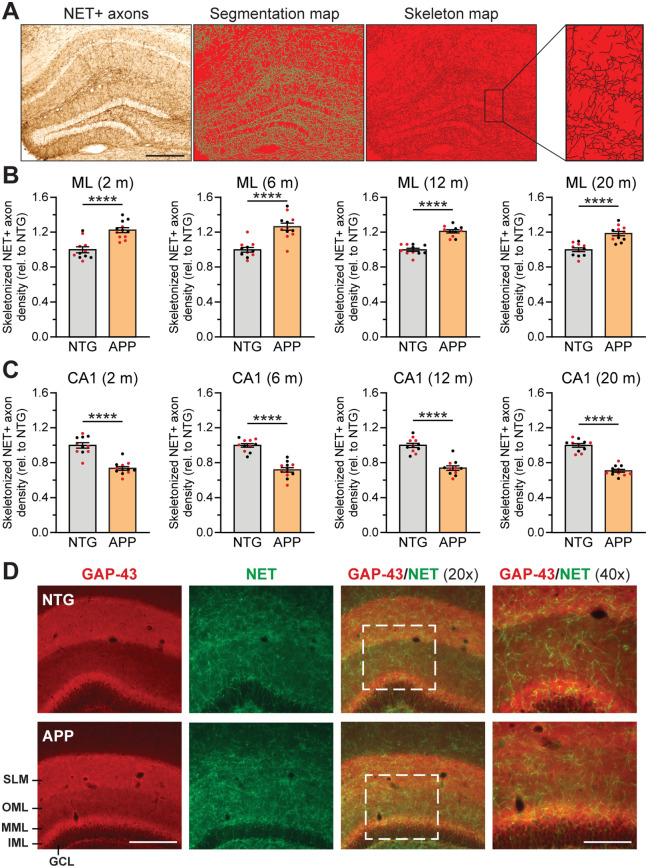
Changes in total axon length underlie alterations in noradrenergic axon density in APP mice. (A) Hippocampus sections immunostained for the norepinephrine transporter (NET) (left) were classified using Trainable Weka Segmentation to produce a binary segmentation map (center) that was skeletonized to generate a skeleton map where axons were represented by traces of uniform thickness (right). Scale bar, 400 µm. (B-C) Density of skeletonized noradrenergic axons in the molecular layer (ML; B) and CA1 (C) of the hippocampus in NTG and APP mice at 2, 6, 12, and 20 months of age, relative to NTG levels (n = 10–12 mice per genotype and age). (D) Norepinephrine transporter (NET) and growth associated protein 43 (GAP-43) double immunostain of the hippocampus in NTG and APP mice. GCL, granule cell layer; IML, inner molecular layer; MML, middle molecular layer; OML, outer molecular layer; SLM, stratum lacunosum-moleculare. Scale bars, 200 µm (20x magnification) or 100 µm (40x magnification). ****, p < 0.0001; unpaired two-tailed t tests or Mann-Whitney tests. Error bars indicate mean ± SEM. Black data points indicate male mice and red data points indicate female mice.

To determine whether the increased noradrenergic axon density in the hilus, ML and SLM of APP mice corresponded to increased total axon abundance (i.e., increased number and/or length) or altered morphology (i.e., increased thickness), we skeletonized the segmentation maps used to calculate axon density, rendering the axons into a “skeleton” of uniform thickness (Fig 2A). We then quantified the area covered by the axon skeleton in the ML and CA1 of NTG and APP mice, where the most robust changes in axon density were observed. We found that the density of the axon skeleton map was increased in the ML (Fig 2B) and reduced in CA1 (Fig 2C) of APP mice. Therefore, the alterations in axon density we measured in Fig 1 most likely represent differences in the abundance of axon segments rather than alterations in axon thickness.

We previously reported that the growth associated protein 43 (GAP-43), a marker of growing neurites and axonal sprouting [56,57], was increased in expression in the ML of APP mice [58]. We therefore examined whether the increase in noradrenergic axon density in the ML might be a consequence of active axonal sprouting. However, co-staining for GAP-43 and NET did not reveal obvious co-labeling in NTG or APP mice (Fig 2D). This result suggests that the increased noradrenergic axon density is not likely to be related to an active sprouting response at the timepoints we examined.

### APP mice do not show overt loss of TH-positive cells or Aβ plaque deposition in the LC

**Fig 3:**
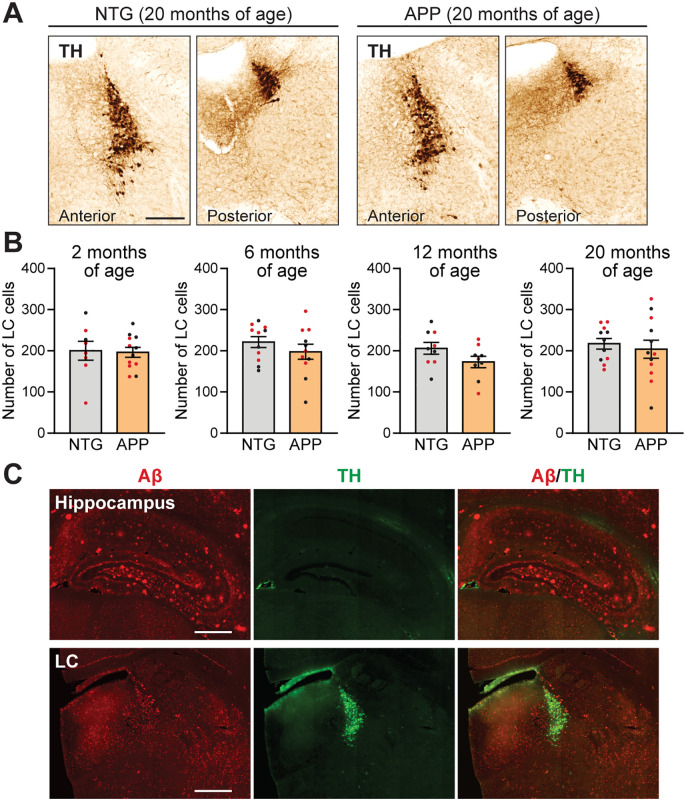
20-month-old APP mice do not have cell loss or Aβ plaques in the LC. **(A)** Tyrosine hydroxylase (TH) immunostain showing LC cells in 20-month-old NTG and APP mice. Scale bar, 250 µm. **(B)** LC cell counts in NTG and APP mice at 2, 6, 12 and 20 months of age showed no significant differences between the genotypes (n = 8–12 mice per genotype and age; unpaired two-tailed t tests). Error bars indicate mean ± SEM. **(C)** Amyloid-β (Aβ) and TH double immunostain of the hippocampus and LC of a representative 20-month-old APP mouse, illustrating the absence of Aβ plaques in the vicinity of the LC. Scale bars, 400 µm. Black data points indicate male mice and red data points indicate female mice.

Death of LC cells has been reported in individuals with MCI and AD [9–14,22], as well as in some mouse models of AD-related neuropathology [32–36]. Given the decreased LC axon density that we observed in multiple areas of the hippocampus of APP mice, we quantified LC cells through aging and disease progression. The mean LC cell counts were not different in APP mice compared to NTG mice at any of the ages examined (Fig 3A, B), indicating that there was no overt loss of noradrenergic cells in the LC of APP mice up to 20 months of age.

In addition to neuronal loss, Aβ plaques can be present in the LC of humans with advanced stages of AD [59], and at least one mouse model of AD neuropathology has shown similar findings [36]. We therefore investigated whether aged APP mice develop Aβ plaques in the LC. Whereas Aβ plaques were abundant in the cortex and hippocampus of 20-month-old APP mice, we did not observe plaques in the LC (Fig 3C). These findings indicate that the changes in noradrenergic axonal density in the cortex and hippocampus of APP mice are independent of neuronal cell loss and Aβ plaque deposition within the LC itself.

### Age-related alterations in noradrenergic axon density follow distinct trajectories in NTG and APP mice

Although individuals with AD exhibit LC cell loss that worsens with disease progression over time [1–6,8], multiple reports indicate that there is no significant LC cell loss associated with normal aging in humans without AD [23,60,61]. However, little is known about whether the innervation of LC projection regions is altered in normal aging. To more fully characterize noradrenergic system function, and dysfunction, in both NTG and APP mice, we compared NET fiber density and LC cell counts within each genotype across aging.

**Fig 4:**
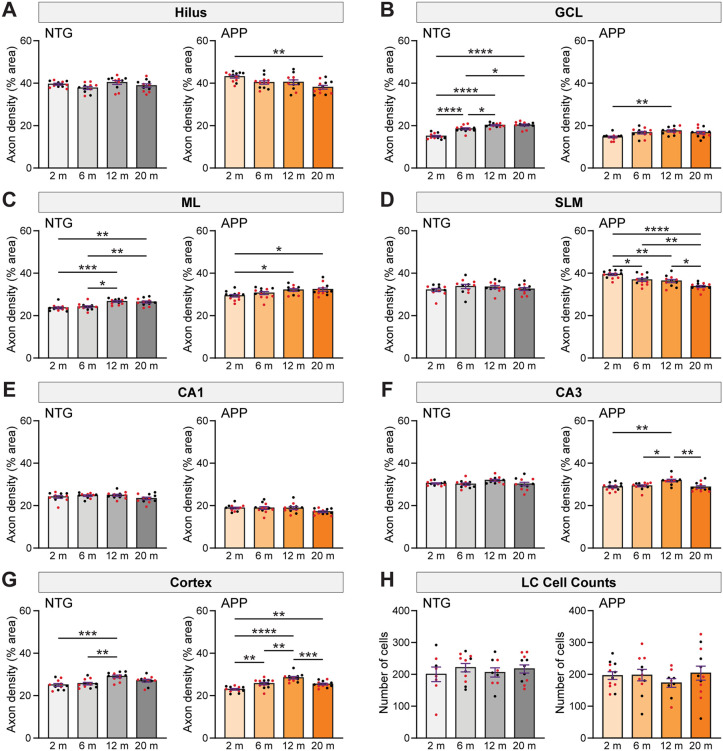
Age-related changes in noradrenergic axon density and LC cell count in NTG and APP mice. (A-F) Age-related dynamics of noradrenergic axon density, measured as percentage area covered by axons, in the hilus (A), granule cell layer (GCL; B), molecular layer (ML; C), stratum lacunosum-moleculare (SLM; D), CA1 (E), and CA3 (F) regions of the hippocampus in NTG (gray) and APP (orange) mice. (G) Age-related progression of noradrenergic axon density in the cortex (n = 10–12 mice per genotype and age). (H) Absence of age-related changes in locus coeruleus (LC) cell counts in NTG and APP mice (n = 8–12 mice per genotype and age). *, p < 0.05; **, p < 0.01; ***, p < 0.001; ****, p < 0.0001; ordinary one-way ANOVA with Holm-Šídák correction for multiple comparisons. Error bars indicate mean ± SEM. Black data points indicate male mice and red data points indicate female mice. ANOVA results are the following: (A) hilus, NTG [F = 2.23, p = 0.0999], APP [F = 5.44, p = 0.0029]; (B) GCL, NTG [F = 34.78, p < 0.0001], APP [F = 4.74, p = 0.0061]; (C) ML, NTG [F = 10.27, p < 0.0001], APP [F = 4.09, p = 0.0122]; (D) SLM, NTG [F = 0.90, p = 0.4487], APP [F = 13.99, p < 0.0001]; (E) CA1, NTG [F = 1.63, p = 0.1981], APP [F = 2.44, p = 0.0771]; (F) CA3, NTG [F = 2.15, p = 0.1086], APP [F = 6.64, p = 0.0009]; (G) cortex, NTG [F = 7.59, p = 0.0004], APP [F = 18.43, p < 0.0001]; (H) LC cell counts, NTG [F = 0.37, p = 0.7717], APP [F = 0.53, p = 0.6616].

We found that NET fiber density in different brain regions changed across aging in different patterns in NTG and APP mice. In the hilus of the dentate gyrus, the density of noradrenergic axons remained constant across age in NTG mice. However, axon density in the hilus of APP mice was reduced at 20 months of age compared to 2 months of age (Fig 4A).

In the GCL, there was a robust increase in noradrenergic axon density across aging in NTG mice, but this aging-related increase was blunted in APP mice (Fig 4B). The ML of both NTG and APP mice exhibited noradrenergic axon density that increased with age (Fig 4C). Notably, NET fiber density was remarkably stable across aging in the SLM of NTG mice but exhibited reductions in APP mice (Fig 4D). Noradrenergic axon density remained constant across aging in CA1 of both NTG and APP mice (Fig 4E), although as seen in Fig 1C, NET fiber density was reduced in APP mice compared to NTG mice at all ages. NTG and APP mice showed a mild age-related increase in axon density in CA3 that peaked around 12 months (Fig 4F), although the change only reached significance in APP mice.

Noradrenergic axon density in the cortex exhibited a modest increase in both NTG and APP mice (Fig 4G) up to 12 months of age, followed by a slight decline at 20 months.

Although NET fiber density differed with aging and with genotype in differing patterns across brain regions, LC cell counts remained stable across aging in both NTG and APP mice, as indicated by an absence of age- or disease-associated loss of cells by 20 months of age (Fig 4H). Overall, we found that whereas some NET axon density dynamics are similar in both NTG and APP mice, the evolution of LC axon density in specific brain regions follows different trajectories in each genotype.

### Magnitude of alterations in noradrenergic axon density is not predicted by amyloid plaque load in APP mice

**Fig 5:**
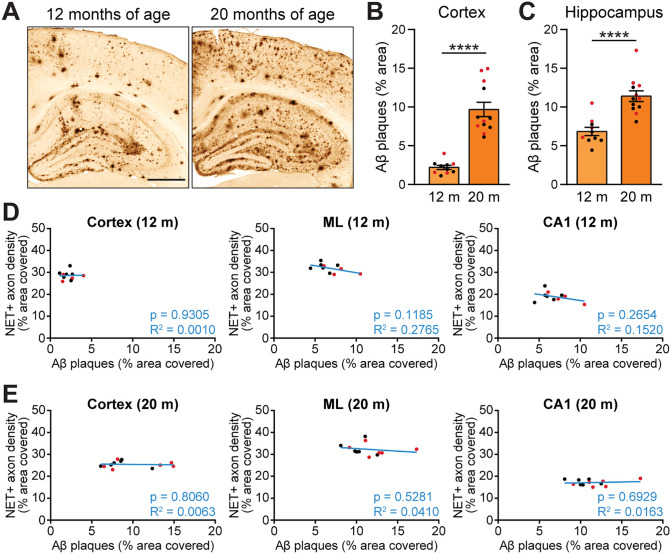
Aβ plaque load does not predict the magnitude of changes in density of noradrenergic axons in APP mice. (A) Aβ plaque burden in hippocampus and cortex of APP mice at 12 and 20 months of age. Scale bar, 500 µm. (B-C) Percentage area covered by Aβ plaques in the cortex (B) and hippocampus (C) of APP mice (n = 10–12 APP mice per age). (D-E) Simple linear regression analyses showing the relationship between Aβ plaque load and noradrenergic axon density in cortex, molecular layer (ML), and CA1 of APP mice at 12 (D) and 20 months of age (E) (n = 10–12 APP mice per age). Cortical noradrenergic axon density was compared to local Aβ plaque load in cortex. Axon density in ML and CA1 was compared to Aβ plaque load in whole hippocampus. ****, p < 0.0001; Mann-Whitney test (B) or unpaired two-tailed t test (C). Error bars indicate mean ± SEM (B-C). Black data points indicate male mice and red data points indicate female mice.

Aβ plaques are a hallmark of AD neuropathology [8], and plaque load corresponds with the degree of neurodegeneration of the LC [62]. Work in mouse models has shown that the deposition of Aβ plaques causes local morphological abnormalities in axons [63] and can result in neurite loss [64] and in axonal sprouting and re-growth [65]. To determine whether Aβ plaque load was associated with the changes in axonal density that we documented in the cortex and hippocampus, we also assessed the area covered by plaques and performed regression analyses on plaque load and noradrenergic fiber density in aged APP mice. Aβ plaque deposition begins around 6 months of age in APP mice of line J20 [47]. We confirmed that Aβ plaque accumulation is robust by 12 months (Fig 5A) and continues to progress with age in the cortex (Fig 5B) and hippocampus (Fig 5C).

We found no relationship between the area covered by Aβ plaques and noradrenergic axon density in the brain regions we examined at either 12 (Fig 5D, S1A Fig) or 20 months of age (Fig 5E, S1B Fig). Some of the observed changes in axon density were already present at 2 months of age, before plaque deposition starts, indicating that plaque accumulation may not be a strong determinant or predictor of the region-specific changes in noradrenergic LC projections in APP mice.

## Discussion

In the present work, we provide a detailed neuroanatomical characterization of the LC-norepinephrine system in an APP mouse model of AD-related neuropathology. We found that APP mice exhibit extensive changes in noradrenergic axon density that are highly region- and age-specific. In all layers of the hippocampus, as well as in the cortex, APP mice showed significant alterations in the density of noradrenergic fibers during at least one of the ages we examined, in comparison with age-matched NTG controls. Some of these changes were present throughout all timepoints from 2 to 20 months of age, whereas others developed at later ages or gradually faded to NTG levels by 20 months of age. Although a number of AD-related features have been found to be sex-dependent, we observed that both male and female APP mice showed similar trajectories for LC-norepinephrine outcome measures in this study.

We observed a decrease in noradrenergic axonal density in the granule cell layer, CA1, and the cortex of APP mice. These results are consistent with previous reports that found reduced noradrenergic fiber density in the dentate gyrus, CA1, and regions of the cortex in other APP mouse models [34,37]. However, we also found that noradrenergic axon density was subtly *increased* in the hilus, molecular layer, and stratum lacunosum-moleculare of APP mice at certain ages. These increases in the density of LC projections are reminiscent of the enhanced axonal growth or sprouting that has been described in individuals with AD and hypothesized to be of a compensatory nature. Several compensatory alterations in the noradrenergic system of individuals with AD have been reported in the literature, including increased activity of the surviving LC cells [29], upregulation of enzymes implicated in norepinephrine synthesis [66], and regionally increased abundance of some adrenoreceptors in certain brain areas [67,68]. Among these alterations is the sprouting of noradrenergic axons, which may be a mechanism aimed at preserving noradrenergic innervation despite dysfunction and loss of LC cells. Noradrenergic axon sprouting has been reported in areas including the hippocampus [25,69] and the prefrontal cortex [28] of individuals with AD.

We sought to confirm the presence of noradrenergic sprouting in the hippocampus of APP mice through analysis of the expression of the growth associated protein 43 (GAP-43), which is expressed in growing neurites [56] and sprouting axons [57]. We previously showed that as early as 4 months of age, APP mice exhibit increased levels of general axonal sprouting in the ML as compared to NTG mice [58]. Here, in co-staining for GAP-43 and NET, we observed high expression of GAP-43 in the interior molecular layer and stratum lacunosum-moleculare of NTG and APP mice (Fig 2D). GAP-43 immunoreactivity appeared elevated in the outer molecular layer of the hippocampus in APP mice with respect to NTG levels (Fig 2D); however, we did not observe clear co-expression of GAP-43 with NET. When we skeletonized NET axons to rule out potential effects of increased thickness of dystrophic neurites, we continued to find increased noradrenergic axon density in APP mice, suggesting that increased fiber abundance (length and/or number) contributes to the increased fiber coverage we observed. We therefore conclude that there is growth of noradrenergic axons in this region. Together, these results confirm the presence of regionally increased noradrenergic axon density in APP mice and suggest that both the loss of LC axons and noradrenergic reinnervation are processes with high spatial and temporal heterogeneity that can affect brain regions differentially, even those in close proximity. Elucidating the factors that underlie this spatiotemporal complexity may contribute to identifying strategies to preserve or restore noradrenergic innervation in disease conditions.

Although LC cell loss is a key feature of the neuropathology of individuals with MCI and AD [9–14,22], we did not find a significant reduction in the number of LC cells in APP mice by 20 months of age. Spontaneous neuronal loss in the LC has been documented in other APP mouse models of AD-neuropathology, including Tg2576 [32], APP/PS1 [33–35], and APP^NL-G-F/NL-G-F^ mice [36]; however, others reports show no significant changes in LC neuronal cell counts in aged mice from lines 5xFAD [41], APP23 [70], hemi- and homozygous PDAPP [71], and APP^NL-G-F/NL-G-F^ [37]. While some of these differences may be a consequence of differing quantification and sample selection methods, they may reflect inherent differences in the characteristics, severity and temporal progression of pathological changes in these mouse models of AD-related neuropathology. We also did not observe Aβ plaques in the LC or neighboring nuclei, a feature of AD neuropathology that is typically only seen in patients with advanced stages of disease [59]. Although there is little information regarding the formation of Aβ plaques in the LC in the various mouse models of AD-related neuropathology, it has been reported to occur in APP^NL-G-F/NL-G-F^ mice at 9 and 12 months of age [36]. The lack of plaques in the vicinity of the LC in J20 APP mice may, again, be a consequence of disparate pathological manifestations and temporal progression in the different models. It is possible that soluble Aβ42, Aβ40, or particular ratios or conformations of Aβ species in the LC may drive LC dysfunction and lead to alterations in axonal projections in other brain regions.

While we did not observe Aβ plaques in the LC, cortical and hippocampal plaques were abundant in APP mice at 12 and 20 months of age. Plaques are known to affect axonal morphology, leading to atrophy, large varicosities and tortuous, swollen processes [63–65]. Sakakibara et al. found dystrophic noradrenergic neurites around Aβ plaques in APP^NL-G-F/NL-G-F^ mice [37], indicating that plaques interact with the LC-norepinephrine system. To further characterize the relationship between Aβ and noradrenergic alterations in APP mice, we examined the relationship between hippocampal and cortical Aβ plaque load and the changes in noradrenergic innervation that we identified. We found no significant associations between the area covered by plaques and norepinephrine axon density in the studied regions. Together with the fact that APP mice showed altered axon density in the hilus, granule cell layer, molecular layer, CA1, and cortex at 2 months of age, long before plaques start to form in this model [47], this finding suggests that Aβ deposits are unlikely to be the primary cause of neuroanatomical pathology of the LC-noradrenergic axons in the hippocampus and cortex. On the other hand, soluble forms of Aβ have neurotoxic effects and can alter neurotransmitter release, trigger neuronal hyperexcitability, and induce synapse loss [72–77], causing alterations before plaques start to form [78]. Although little is known about interactions between soluble Aβ species and the LC-norepinephrine system, given the fact that APP mice do not express mutant tau protein and that some of the observed alterations precede plaque deposition, increased levels of soluble Aβ may be critical in driving the observed changes in APP mice. Future studies that measure levels of soluble Aβ42 and Aβ40 in the cortex and hippocampus, and perhaps examine various conformations of Aβ oligomers, could provide further insight into specific Aβ conformations that are most closely associated with alterations in noradrenergic axons. For example, J20 APP mice exhibit similar Aβ42/40 ratios in the hippocampus and cortex, but much higher levels of Aβ oligomers in the hippocampus than in the cortex [79], suggesting that particular species of Aβ may relate more closely with specific outcome measures. Our findings demonstrate that APP mice display highly age- and region-specific changes in noradrenergic fiber density in the hippocampus and cortex in the absence of cell loss and Aβ plaques in the LC. These alterations reflect actual changes in axon length and/or number, as opposed to changes in axon shape or thickness. Norepinephrine axon density alterations preceded Aβ plaque formation and exhibited no relationship with plaque load in the hippocampus and cortex. This work adds to the growing body of knowledge about noradrenergic pathology in the context of AD and will help to put into context previous and future studies regarding this topic.

## Supporting information

S1 Fig Aβ plaque load does not predict the magnitude of changes in density of noradrenergic axons in hilus, granule cell layer, stratum lacunosum-moleculare or CA3 of APP mice. (PDF)

S1 Datasheet Extended statistics containing sex-segregated and sex-combined analyses. (XLSX)
