# Supplementary material for: Noradrenergic innervation across brain regions is altered by aging and by disease progression in a mouse model of Alzheimer’s disease neuropathology
Source: PLoS One. 2026 Feb 18;21(2):e0340611. doi: 10.1371/journal.pone.0340611 (PMC12915906; doi:10.1371/journal.pone.0340611)
Supplement: S1 Fig — (PDF) [file pone.0340611.s001.pdf]

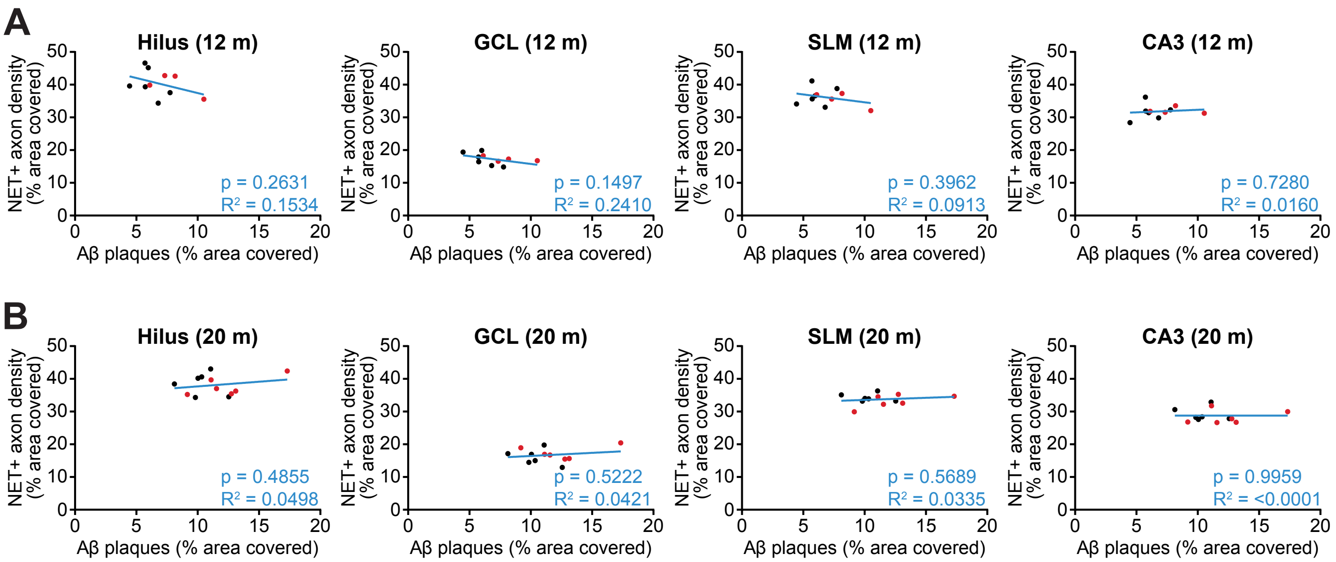

S1 Fig. Aβ plaque load does not predict the magnitude of changes in density of noradrenergic axons in hilus, granule cell layer, stratum lacunosum-moleculare or CA3 of APP mice. (A-B) Simple linear regression analyses showing the relationship between Aβ plaque load and noradrenergic axon density in the hilus, granule cell layer (GCL), stratum lacunosum-moleculare (SLM), and CA3 of APP mice at 12 (A) and 20 months of age (B) (n = 10–12 APP mice per age). Axon density was compared to Aβ plaque load in whole hippocampus. Black data points indicate male mice and red data points indicate female mice.
